# Manifestations of Ovarian Cancer in Relation to Other Pelvic Diseases by MRI

**DOI:** 10.3390/cancers15072106

**Published:** 2023-03-31

**Authors:** Charis Bourgioti, Marianna Konidari, Lia Angela Moulopoulos

**Affiliations:** Department of Radiology, School of Medicine, National and Kapodistrian University of Athens, Aretaieion Hospital, 76 Vas. Sofias Ave., 11528 Athens, Greece

**Keywords:** ovarian cancer, malignancy, benign tumors, mimickers, MR

## Abstract

**Simple Summary:**

Characterization of an adnexal mass may be challenging since there are several benign and malignant pelvic conditions with similar appearances on imaging. The aim of this study is to comprehensively review discriminative MRI features of common and uncommon adnexal masses in order to help radiologists more accurately diagnose ovarian cancer. Imaging findings of ovarian tumors in specific settings, including adolescence and pregnancy, are also discussed.

**Abstract:**

Imaging plays a pivotal role in the diagnostic approach of women with suspected ovarian cancer. MRI is widely used for preoperative characterization and risk stratification of adnexal masses. While epithelial ovarian cancer (EOC) has typical findings on MRI; there are several benign and malignant pelvic conditions that may mimic its appearance on imaging. Knowledge of the origin and imaging characteristics of a pelvic mass will help radiologists diagnose ovarian cancer promptly and accurately. Finally, in special subgroups, including adolescents and gravid population, the prevalence of various ovarian tumors differs from that of the general population and there are conditions which uniquely manifest during these periods of life.

## 1. Introduction

### 1.1. Data Search

Literature search for this narrative review was conducted using MEDLINE (PubMed) Library. Applied key words included the following terms: ovarian neoplasms; ovarian masses; ovarian malignancy; probability of malignancy; ovarian benign tumors; ovarian tumor mimickers; MRI. The search period extended from July 1997 to November 2022. Studies with prospectively and retrospectively collected data and review articles including systematic meta-analyses were considered. All authors agreed on the following inclusion criteria: acceptable methodology, adequate data collection, use of clear diagnostic evidence, sufficient statistical analysis, and reproducibility of results. A total of 122 studies was considered eligible and included in this review.

### 1.2. Epidemiology

The ovary is made of a variety of different cell types and is, therefore, the site of diverse tumors. Ovarian neoplasms are divided into four main categories depending on the cell of origin: epithelial, germ cell, sex cord–stromal tumors, and metastases. Epithelial neoplasms account for the majority of primary ovarian tumors, followed by germ cell tumors [1]. Most of these tumors are benign or borderline, however, malignant epithelial neoplasms are a serious public health issue since they often manifest as advanced stage disease. Ovarian cancer is the second most frequent gynecologic malignancy. With a total of 313,959 new cases recorded globally in 2020, an age-standardized incidence rate of 6.6/100,000 and an age-standardized mortality rate of 4.2/100,000 women/year, ovarian cancer is the fifth leading cause of cancer death in women [2]. Most ovarian cancer deaths are caused by epithelial cell carcinomas, specifically high-grade serous cystadenocarcinoma, which is the most common subtype and accounts for approximately 64% of all epithelial ovarian cancers.

#### Pitfalls and Diagnostic Challenges

**Figure 1:**
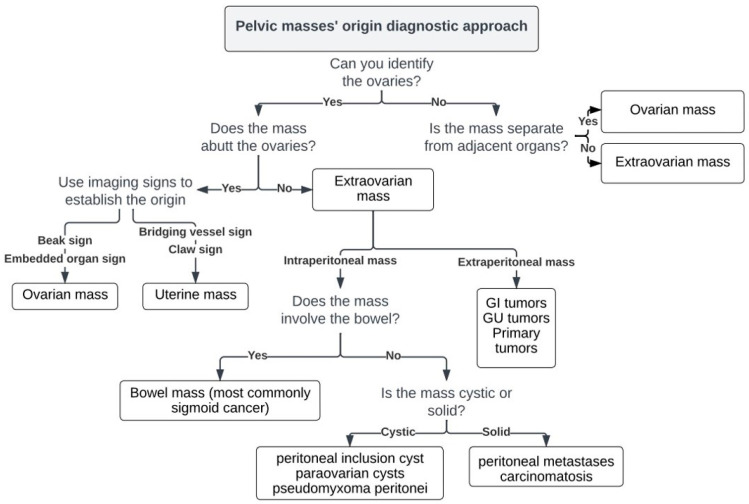
Imaging approach of pelvic masses’ origin.

Imaging plays a significant role in the diagnostic approach of women with suspected ovarian cancer. The two main challenges faced by radiologists when assessing a suspicious pelvic mass include (a) confirming its origin (ovarian or extraovarian) and (b) characterizing its nature (benign or malignant and, if possible, the most likely diagnosis). Both these are important for narrowing the differential diagnosis and helping decision making (Figure 1). All imaging modalities including Ultrasound (US), Magnetic Resonance Imaging (MRI), and Computed Tomography (CT) can be used to assess pelvic masses, each having different advantages and offering complementary information.

Pelvic US is the modality of choice for the initial evaluation of an adnexal mass and, in most cases, can accurately characterize the ovarian lesion as benign or malignant. However, a varying range of ovarian lesions (5–40%), depending on the experience of the sonographer, may remain indeterminate after initial evaluation [3,4,5,6,7].

### 1.3. Origin and Characterization of a Pelvic Mass

To establish an ovarian origin of a pelvic mass, the following stepwise approach may be used:

- Identify the ovaries.

An oblique coronal T2-weighted sequence parallel to the endometrium, is particularly useful in determining ovarian versus extraovarian origin of a pelvic mass. If a normal ipsilateral ovary is identified, the mass cannot be of ovarian origin. Note that when finding the ovary on MR images is difficult, e.g., in postmenopausal women, you may follow the gonadal vessels anterior to the psoas muscle and all the way to the ovary [8]. This is facilitated on contrast-enhanced images of the pelvis. When a normal ovary cannot be identified (e.g., in large pelvic masses), but the mass appears separate from other pelvic organs (bladder, bowel, or uterus) and the gonadal vessels run into it, then an ovarian origin is highly likely [9]. However, advanced ovarian cancer is often seen to invade adjacent pelvic structures, in which case establishing the primary site can still be difficult.

- Describe relationship to the ovary.

Mass abutting the ovary: If a mass abuts the ovary, this does not necessarily indicate an ovarian origin, since it can as well originate from adjacent pelvic structures. Several previously described imaging signs are useful for determining the ovarian origin of a pelvic mass, such as the presence of the beak sign, created by the ovarian tissue partly enveloping the mass and forming sharp angles with it and the embedded sign, when the ovary appears to be engulfed by the tumor [10]. However, the bridging vessel sign as well as the claw sign are indicative of uterine origin. The bridging vessel sign is present when vessels extend between the uterus and the mass, while the claw sign is present when uterine tissue is draped around the mass; both signs are commonly seen in cases of uterine leiomyomas [11].

Ovary not involved: If the ovary is not involved but the mass is intraperitoneal, one should consider the bowel as the site of origin, particularly the sigmoid colon. If the mass is intraperitoneal but separate from the ovary or bowel, it is important to determine its nature. Cystic masses may be of benign nature, such as peritoneal inclusion and para-ovarian cysts or they may be malignant, such as pseudomyxoma peritonei. Solid masses more commonly include peritoneal metastases or carcinomatosis. Extraperitoneal masses most often result from direct extension of gastrointestinal (rectal) or genitourinary (bladder, uterine, cervical) tumors. However, primary tumors, mainly of mesenchymal or neurogenic origin, can also occur in the extraperitoneal spaces.

Characterization of an ovarian mass is based on a combination of clinical information (i.e., age, elevated levels of tumor markers etc.) and imaging findings.

**Figure 2:**
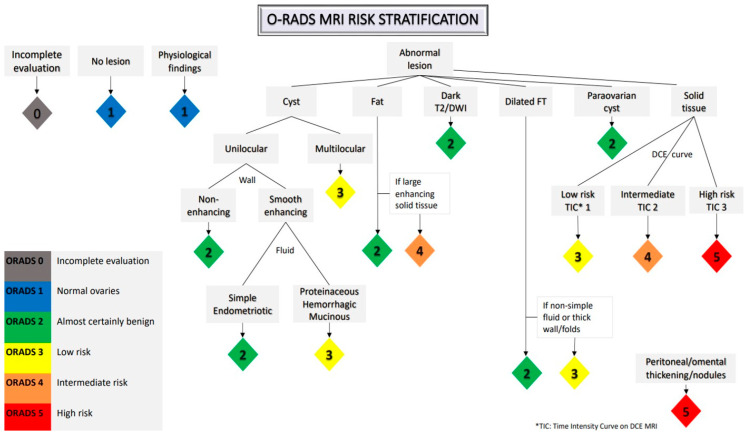
Ovarian-adnexal reporting and data MRI risk stratification system.

MRI is a problem-solving tool for preoperative characterization and subsequent risk stratification of indeterminate adnexal masses [12,13,14,15]. MRI provides better contrast resolution between different soft-tissue components (e.g., fat, hemorrhage, fibrous tissue) without the use of ionizing radiation [16]. The Ovarian-Adnexal Reporting and Data System (O-RADS) MRI is a recently developed and validated scoring system which is now proposed for risk assignment of sonographically indeterminate adnexal masses. This MRI scoring system includes six categories with different risks of malignancy and it is based on MRI features with high positive and high negative predictive values in distinguishing benign from malignant masses [17] (Figure 2).

Computed Tomography (CT) is recommended for ovarian cancer staging as it provides excellent spatial resolution and very short examination times [18]; however, it does not assist the characterization of an adnexal mass apart from identifying fat and calcifications (mostly in cases of teratomas).

### 1.4. Importance of Accurate Diagnosis in Treatment Decisions

Accurate differential diagnosis of pelvic masses based on imaging is crucial since it largely affects the clinical management and treatment decisions.

Establishing the site of origin and providing the most likely diagnosis of a pelvic mass can help determine whether the patient should be referred to a gynecologist (benign lesions), gynecologic oncologist (malignancy), or other specialist (suspected metastases to the ovary or non-gynecologic tumor), and which is the best treatment option.

Regarding management of ovarian neoplasms, benign ovarian tumors may be followed-up or treated with conservative surgery. Malignant epithelial ovarian neoplasms are most often present at an advanced stage with peritoneal dissemination. Cytoreductive surgery followed by adjuvant chemotherapy is the treatment of choice. Primary neoadjuvant chemotherapy may be applied in cases of extensive peritoneal disease, followed by interval debulking surgery [19]. Fertility-sparing surgery (i.e., unilateral salpingo-oophorectomy (USO) with preservation of the uterus and contralateral ovary), may be an option in cases of malignant germ cell tumors of any stage, sex-cord stromal and borderline histology, or even early-stage epithelial carcinomas [20,21].

In general, the clinical management is mainly influenced by the tumor’s nature (benign or malignant), histologic subtype and stage, since these are the main prognostic factors [22]. Imaging can provide useful information for all the above. The established US and MRI scoring systems are helpful tools in stratifying the risk of malignancy and discriminating between benign and malignant tumors. Several US-based models have been employed to differentiate benign from malignant adnexal masses, such as the IOTA group Simple Rules or ADNEX model, the Gynecologic Imaging Reporting and Data System (GI-RADS) and more recently the O-RADS US risk stratification and management system by the ACR Ovarian-Adnexal Reporting and Data System Committee [23,24,25,26]. Regarding different histologic subtypes, although some of them have distinguishing imaging features, findings are often overlapping and nonspecific. Even though final diagnosis requires histopathologic analysis, specific features and typical imaging findings of ovarian neoplasms may limit the differential diagnosis. Finally, accurate staging of ovarian cancer is vital, given the fact that the extent of disease as well as residual disease after surgery affect prognosis and survival. CT and DWI MRI both have high sensitivity in depicting peritoneal disease and can provide valuable information to clinicians for selecting the best treatment option [27].

### 1.5. Special Subgroups

#### 1.5.1. Adolescence

Ovarian tumors in children and adolescents differ from those in adults, regarding incidence, histology, and presentation. In general, ovarian neoplasms in childhood are uncommon and usually benign, with germ cell tumors (GCTs) being the most common type, followed by epithelial and stromal cell tumors [28]. In many cases, they present with abnormal hormonal secretion, unusual sexual development, and increased serum tumor markers (i.e., AFP in immature teratoma and yolk sac tumor, β-hCG in dysgerminoma, CA-125 in epithelial neoplasms). Imaging can help discriminate between benign and malignant ovarian neoplasms since malignant masses usually appear predominantly solid, more heterogeneous, and larger than benign tumors. In a recent study MRI showed 100% specificity and sensitivity in differentiating benign and malignant pediatric ovarian tumors [29].

#### 1.5.2. Pregnancy

Adnexal masses are common during pregnancy, discovered in 0.1 to 2.4% of pregnant women. Most adnexal lesions in the gravid population are benign, mostly functional, with only 1–5% being malignant [30,31]. Germ cell tumors, sex cord (stromal) tumors, and borderline tumors are the most common malignant ovarian neoplasms in the gravid population [32], whereas epithelial ovarian cancer accounts for only 35% of ovarian cancers in pregnancy [33].

Evaluation of adnexal masses in the gravid population is challenging because they may undergo morphologic changes due to the altered hormonal status and may demonstrate features that can mimic malignancy on imaging. The most common pregnancy-related adnexal masses are the corpus luteum of pregnancy and the theca lutein cyst; both are expected to resolve after gestational week 18, although a few may persist until after delivery [34]. Other conditions that can mimic malignancy at imaging are decidualized endometriomas and leiomyomas with red degeneration. Decidualized endometriomas are the result of progesterone action and increased glandular endometrial secretion and are characterized by increased blood flow and intraluminal papillary projections [35]. Red degeneration is a subtype of hemorrhagic infarction of leiomyomas that often occurs during pregnancy due to venous thrombosis within the periphery of the mass or rupture of intra-tumoral arteries [36].

## 2. Typical and Atypical Findings of Ovarian Cancer and Mimickers on MRI

### 2.1. Borderline and Malignant Neoplasms

Imaging findings indicative of borderline or malignant tumors include mural nodules, papillary projections, enhancing solid tissue (except those of fatty or fibrous nature), thickened, irregular walls or septa (i.e., diameter > 3 mm and highly vascular), and necrosis. Large size and the involvement of lymph nodes or peritoneal dissemination may suggest a borderline or malignant tumor [37].

The epithelial subtype accounts for most borderline and malignant ovarian neoplasms. According to the 2014 WHO classification, epithelial ovarian neoplasms include serous, mucinous, endometrioid, clear cell, seromucinous, Brenner, and undifferentiated tumors [1]. Serous malignant tumors can be further classified into low- and high-grade tumors.

#### 2.1.1. High-Grade Serous Cystadenocarcinoma (HGSC)

HGSC is the most common histological type of ovarian malignancy accounting for almost half of ovarian cancer cases [2]. It usually affects postmenopausal women (mean age: 60 years), and most often presents at an advanced stage. Serum CA-125 levels are elevated in up to 90% of patients with HGSC [38].

**Figure 3:**
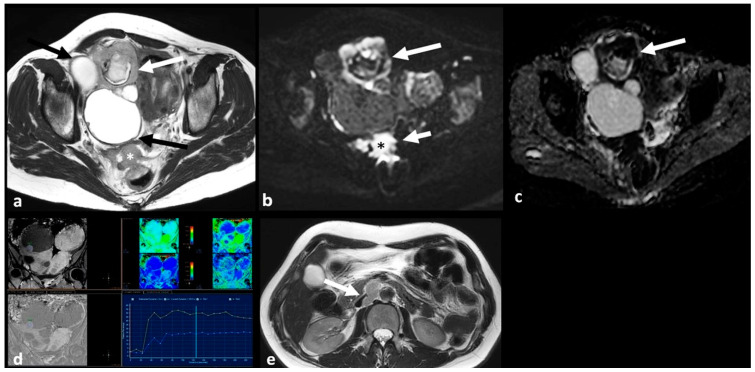
High grade serous cystadenocarcinoma with peritoneal and lymph node metastases. Axial T2 weighted image (**a**) shows a multiloculated right ovarian cystic mass (black arrows) with a large solid component (white arrow). The solid portion (white arrow) demonstrates high signal on axial 1200 b value DWI (**b**) and low signal on ADC map (**c**). DCE image analysis (**d**) demonstrates an intermediate-risk time intensity curve of the solid tissue (TIC type 2, orange line: myometrium, blue line: solid tissue). Axial T2 weighted image of the upper abdomen of the same patient (**e**) shows right paraaortic lymphadenopathy (white arrow). Shown also is a large peritoneal implant in the pelvis (asterisk in (**a**); asterisk and short arrow in (**b**)).

HGSC usually manifests as bilateral (58% of cases), predominantly cystic masses with differing amounts of solid tissue. A few of such tumors are entirely solid. The solid component often demonstrates restricted diffusion and intense enhancement on dynamic contrast enhanced images with type 3 Time Intensity Curve (TIC) (i.e., initial slope greater than myometrium and marked increase in signal intensity with a plateau or washout) [17,39]. It usually presents with extraovarian disease at diagnosis, including peritoneal dissemination, pelvic organ invasion, ascites, and lymphadenopathy [40] (Figure 3).

#### 2.1.2. Serous Borderline Neoplasms and Low-Grade Serous Cystadenocarcinoma

The most often diagnosed borderline tumor is a serous borderline neoplasm (65–70%). It is most common in young women (mean age: 42 years) compared to their high-grade counterparts and has excellent overall prognosis [41].

**Figure 4:**
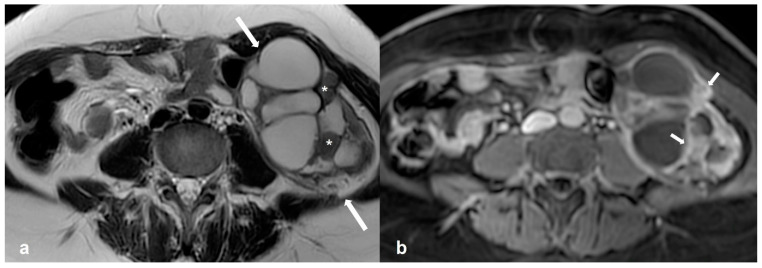
Low grade serous cystadenocarcinoma. Axial T2 weighted image (**a**) shows a multiloculated, predominantly cystic mass at the left ovary (white arrows) with solid tissue (asterisks) showing avid enhancement on the T1 weighted FS CE image ((**b**), white arrows).

Low-grade serous cystadenocarcinoma (LGSC) is uncommon, accounting for only 2.5% of ovarian malignancies. Interestingly, it can arise from and co-exist with a non-invasive serous borderline component. Compared to borderline neoplasms, women with LGSC often present at a later stage and have a poorer prognosis since LGSC is platinum-resistant [42] (Figure 4).

**Figure 5:**
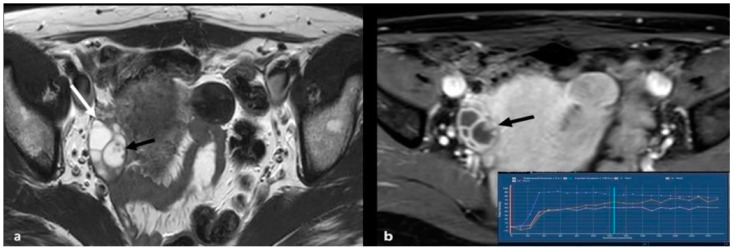
Serous epithelial borderline ovarian tumor. Axial T2 weighted image (**a**) shows a multiloculated cystic lesion at the right ovary (white arrow). A small, papillary projection (black arrow) is present, with avid enhancement on the T1 weighted FS CE image ((**b**), black arrow). An intermediate-risk time intensity curve (TIC type 2) was detected on DCE image analysis (inset in b, blue line: myometrium, orange/pink lines: papillary projection).

Tumors of borderline and low-grade serous histology, manifest as multilocular cystic masses, with solid tissue in the form of papillary projections or mural nodules and rarely, surface nodules. The solid elements enhance after intravenous contrast administration, usually with type 2 TIC on DCE images (i.e., initial slope less than myometrium, moderate increase in signal intensity with a plateau or washout). Compared to their mucinous counterparts, borderline and low-grade serous neoplasms are more often bilateral (around one-third of serous borderline tumors and the majority of LGSC) with an increased number of papillary projections [43] (Figure 5).

Tip: Epithelial tumors of low malignant potential exhibit an increased number of papillary projections. Although these can also be found in invasive carcinomas, the latter usually have a dominant solid component. According to a previously published study with CT and MRI, papillary projections were detected more frequently in ovarian tumors of low malignant potential (67%) followed by malignant (38%) and benign (13%) neoplasms [44].

#### 2.1.3. Mucinous Borderline Neoplasms and Mucinous Cystadenocarcinoma

Tumors of mucinous histology account for 10–15% of all ovarian neoplasms; malignant mucinous tumors are quite rare [45]. In general, mucinous borderline neoplasms involve younger women than their serous counterparts and they are associated with an excellent outcome [41]. Conversely, mucinous adenocarcinoma, which accounts for 9.4% of all invasive epithelial tumors, has the poorest prognosis of all ovarian malignancies, with low survival rates [46].

**Figure 6:**
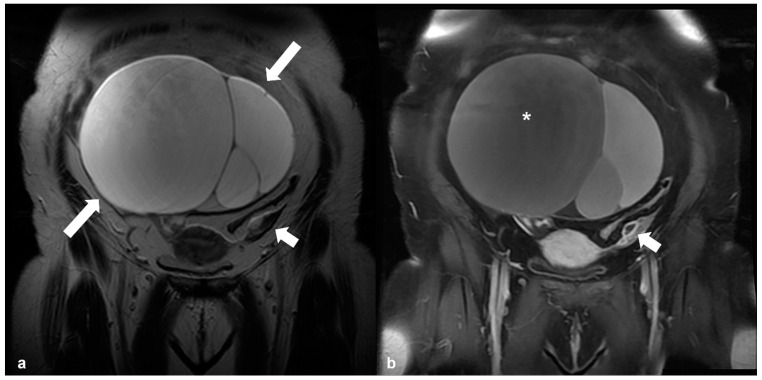
Mucinous cystadenoma. T2 weighted image (**a**) in the coronal plane, shows a large multilocular cystic mass originating from the right ovary (arrows). Corresponding T1 CE FS image (**b**) shows different signal intensity of various compartments (asterisk), the so-called ‘stained-glass’ appearance. Shown also is the normal left ovary (short arrow in (**a**,**b**)).

Mucinous neoplasms are usually unilateral, confined to the ovary, and larger when diagnosed compared with serous tumors. At imaging, they appear as multilocular, predominantly cystic masses. The signal intensity of the locules may vary on MRI, because of different mucin content, the so-called stained-glass appearance [45] (Figure 6).

Papillary solid elements, described in serous neoplasms, are not a common finding [44]. Intramural (often linear) calcifications are present in about one-third of cases [47]. Discrimination between benign and borderline mucinous neoplasms can be challenging. Features indicative of a borderline histology, include increasing size and number of locules, fluid content with high T1 or low T2 signal intensity, and mural nodules or septa > 5 mm thick [48]. Typical features of mucinous cystadenocarcinomas include large solid component and size > 10 cm, with internal smaller loculi (i.e., a honeycomb appearance) [49]. Rupture of a mucinous cystadenocarcinoma into the peritoneal cavity results in pseudomyxoma peritonei (PMP); however, in most cases, PMP is caused by rupture of a mucinous neoplasm of the appendix and, less frequently, rupture of a primary ovarian mucinous tumor [50].

#### 2.1.4. Endometrioid Carcinoma and Clear Cell Carcinoma

Endometrioid and clear cell ovarian carcinomas are typically invasive and aggressive, although typically they are low grade neoplasm [51]. Both commonly affect women in their fifties in the 5th decade and present at an early stage, leading to better clinical outcomes [46]. There is an increased incidence of these tumors with Lynch syndrome [52], endometrial carcinoma, and endometriosis (39% and 41%, respectively) [53].

**Figure 7:**
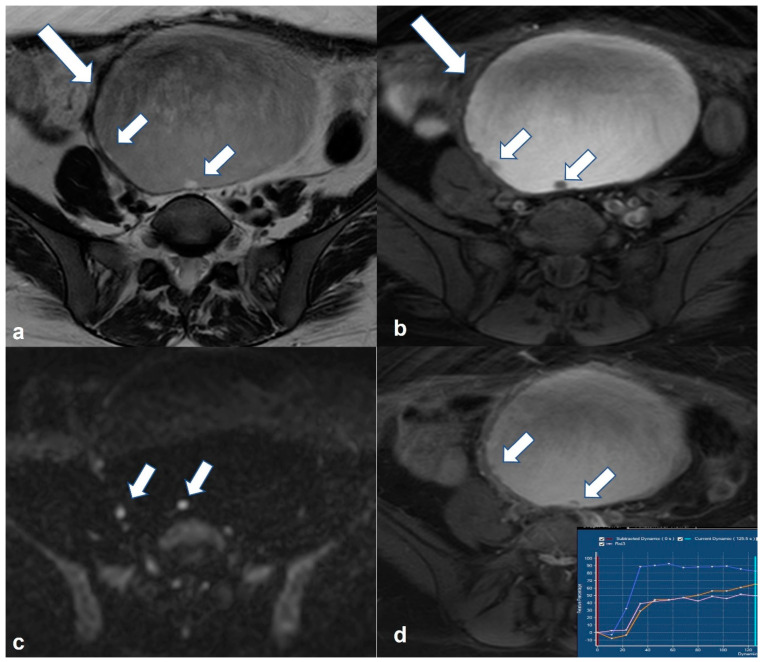
Endometrioid borderline ovarian tumor Axial T2 weighted image (**a**) shows a cystic mass (large arrow) originating from the right ovary with hemorrhagic content on corresponding axial T1 weighted FS image (**b**). There are small mural nodules in the right posterolateral aspect of the lesion (small arrows in a and (**b**)) with restricted diffusivity on corresponding axial high b value DWI image (small arrows in (**c**)). Note the enhancement of the nodules on the corresponding axial T1 weighted FS CE image (small arrows in (**d**)). DCE analysis showed a type 2 time–intensity curve (inset in (**d**), blue line: uterus; yellow/pink line: nodules).

Imaging findings of endometrioid and clear cell carcinomas are nonspecific. High-grade endometrioid carcinoma can be indistinguishable from HGSC. Both are typically present as a mass with varying solid and cystic parts, usually more solid than serous and mucinous neoplasms, often with evidence of hemorrhage. When they develop in an endometrioma, diagnosis may be suggested by the presence of an enhancing mural nodule within an otherwise typical endometrioma (particularly with large endometriomas in women > 45 years) [54,55] (Figure 7). Loss of T2 shading on MRI may be another sign of malignancy due to dilution of hemorrhagic contents by non-hemorrhagic fluid produced by the malignant tissue [55].

**Figure 8:**
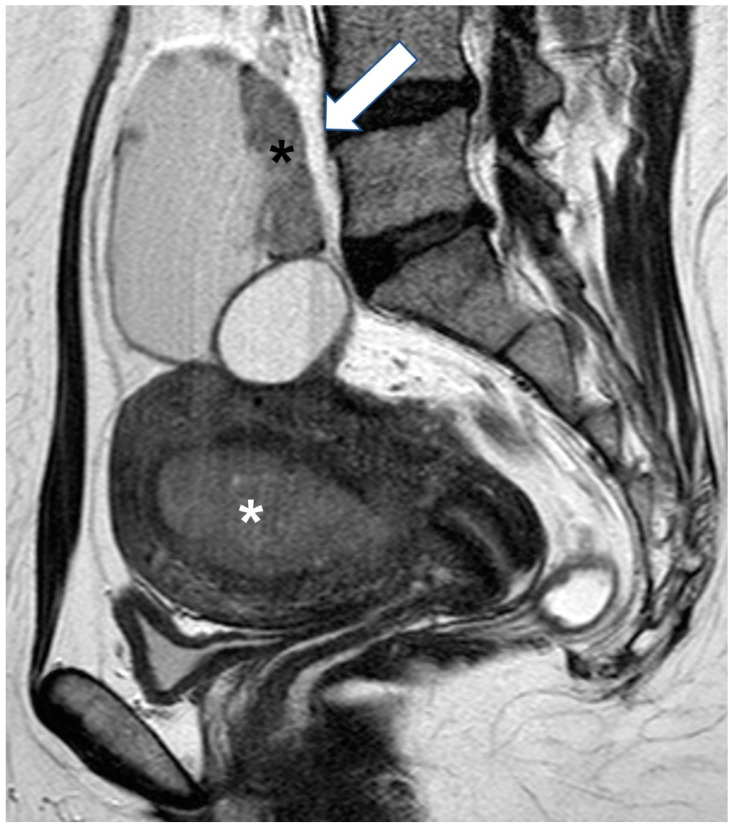
Synchronous ovarian and endometrial endometrioid adenocarcinoma. Sagittal T2 weighted image shows a soft-tissue mass occupying the endometrial cavity (white asterisk). Note a solid and cystic mass of the right ovary (arrow) with the solid component (black asterisk) exhibiting similar signal intensity to that of the endometrial mass.

Tip: Concurrent with ovarian tumor endometrial thickening or mass may suggest endometrioid carcinoma [56] (Figure 8). Thromboembolic episodes (which may occur as a complication in 1/3 of clear cell tumors), hypercalcemia and a mostly solid ovarian mass should suggest a diagnosis of clear cell carcinoma [57,58].

Tip: Although rare, endometrioid carcinoma is the most common malignancy arising within an endometriotic cyst, followed by clear cell carcinoma.

### 2.2. Benign Tumors That Can Mimic EOC

#### 2.2.1. Cystadenofibroma

Adenofibromas and cystadenofibromas are rare epithelial–stromal neoplasms most often of the serous subtype that are almost always benign. They are usually discovered incidentally but sometimes they may cause symptoms related to hormone production; most commonly vaginal bleeding due to excessive estrogen secretion. They rarely have borderline or malignant features, but even the benign subtypes can mimic malignant neoplasms at imaging [59].

**Figure 9:**
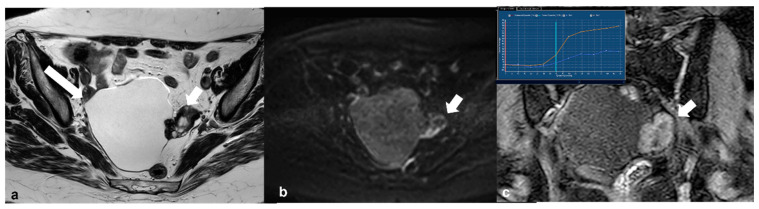
Cystadenofibroma. Axial T2 weighted image (**a**) shows a right-sided ovarian cystic mass (long arrow) with a peripheral solid component of very low signal intensity (short arrow). Corresponding axial high b value (1200) diffusion weighted image (**b**) shows no restricted diffusion of the solid tissue (arrow). Dynamic contrast-enhanced image (**c**) demonstrates avid enhancement of the solid part of the lesion (arrow) and a type 2 time–intensity curve (yellow line: uterus; blue line: lesion in inset).

Typically, they appear as mixed solid and cystic masses, often with papillary projections. At MRI, the solid components which correspond to the fibrous stroma are characteristically quite hypointense on T2 weighted images, of lower signal intensity compared to muscle, often with internal cysts and minimal enhancement [60]. The low T2 signal of the septa, together with the high T2 signal of the cystic spaces give the tumor a characteristic ‘black sponge’ appearance (Figure 9). If the fibrous component displays higher T2 signal intensity or stronger enhancement, based on occasional case reports, the extremely rare malignant cystadenocarcinofibroma may be considered [61].

#### 2.2.2. Fibrothecoma

Fibromas, thecomas, and fibrothecomas are benign stromal ovarian tumors. Usually, they are incidentally found and are asymptomatic, however, occasionally, they may manifest with abdominal pain, in the event of torsion, or with abnormal vaginal bleeding due to estrogen secretion from the thecoma component [62,63].

**Figure 10:**
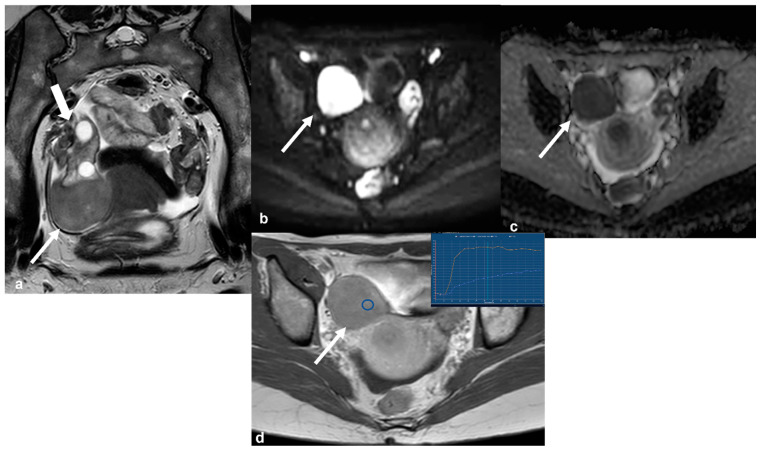
Cellular fibrothecoma. T2 weighted image in the axial oblique plane (**a**) demonstrates a rather low signal intensity lesion (long white arrow) attached to the lower pole of the right ovary (short white arrow). Corresponding axial high b value (1200) diffusion weighted image (**b**) and ADC map (**c**) show restricted diffusion of the lesion (arrow in (**b**,**c**)). The lesion shows avid contrast uptake on axial CE T1 weighted image ((**d**), arrow). DCE analysis of the lesion shows a type 1 time–intensity curve (inset in (**d**), blue line: lesion; orange line: uterus; blue circle: region of interest (ROI) within the lesion).

Typically, on MRI, they present as solid ovarian masses with low T2 signal intensity relative to muscle. However, T2 signal intensity may be higher when oedema or cystic degeneration co-exist (most often in thecomas) [64]. In most cases, they demonstrate low signal intensity both on DWI and ADC maps (known as the T2 blackout effect) due to the presence of fibrous tissue [65,66]. On DCE, they demonstrate minimal enhancement initially, which increases on delayed images; this perfusion pattern corresponds to TIC type 1 (i.e., mild and gradual increase in signal over time with no well-defined shoulder and no plateau) [66] (Figure 10).

Tip: Functioning thecomas and cellular fibromas may show restricted diffusivity due to higher cellularity [67].

Tip: The degree of contrast enhancement differs with the amount of fibrous tissue. While the fibrous tissue demonstrates delayed weak enhancement on DCE, the theca cells are highly vascularized. That explains why thecomas may demonstrate a TIC type 2 or 3 compared to the typical TIC type 1 of fibrothecomas on DCE [17,68].

Fibrothecomas are often indistinguishable from other fibrous tumors such as Brenner tumors which are also hypointense on T2 weighted images. Fibrothecomas are usually of larger size than Brenner tumors and calcifications are not so common, although dense calcifications may sometimes be present [64,69].

**Figure 11:**
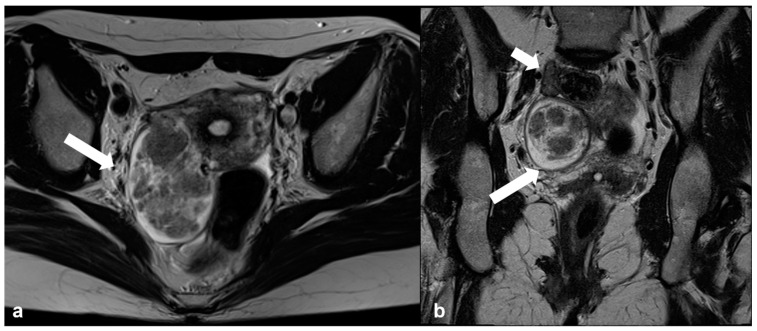
Degenerative pedunculated leiomyoma mimicking ovarian lesion. Axial T2 weighted image (**a**) shows a large inhomogeneous mass within the right broad ligament (arrow). Coronal oblique T2 weighted image (**b**) of the same patient shows the right ovary (short arrow) separate from the mass (long arrow).

In some cases, they may be misinterpreted for a pedunculated leiomyoma, but this usually displays more intense homogeneous enhancement similar to that of the myometrium and possibly, the bridging vessel sign [11] (Figure 11).

#### 2.2.3. Pelvic Inflammatory Disease—Tubo-Ovarian Abscess (TOA)

Patients with PID and tubo-ovarian abscesses, usually present with typical clinical symptoms such as fever and abdominal pain, and the diagnosis is made with transvaginal US. However, in some cases, especially in older patients, unusual causes, or chronic stage, the imaging findings may be indeterminate and mimic malignancy.

**Figure 12:**
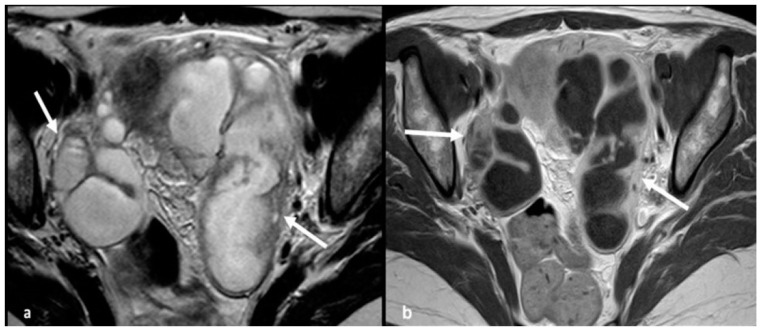
Acute pelvic inflammatory disease. Axial T2 weighted image (**a**) shows large tubular pelvic structures bilaterally (arrows) with thick, smooth walls and incomplete folds, containing non-simple fluid, consistent with dilated tubes. Corresponding T1 weighted contrast-enhanced image (**b**) demonstrates enhancement of the tubal wall but no internal enhancing solid tissue.

MRI usually demonstrates a low T1 signal-intensity cystic mass with heterogeneous high signal intensity on T2-weighted images depending on the content and protein concentration [70]. T2-shading at the periphery of the cyst and a hyperintense halo on T1-weighted images have also been described and may help the differential diagnosis [71]. Typical MRI features of pyosalpinx include fluid-filled tubular structures with enhancing, thick walls usually adjacent to and inseparable from a TOA (Figure 12).

The presence of gas is pathognomonic of a tubo-ovarian abscess although only seen in 22–38% of cases [72]. There is infiltration of the perilesional fat and in chronic cases, adhesions. In acute PID, diffusion restriction is commonly seen due to highly viscous internal proteinaceous material. However, diffusion restriction may be absent in chronic abscesses or TOA after antibiotic treatment [73].

### 2.3. Rare Ovarian Neoplasms That Can Mimic EOC

#### 2.3.1. Brenner Tumor

Brenner tumors are uncommon neoplasms of epithelial-stromal origin, almost always benign with only a few reports of borderline and malignant histology [74].

**Figure 13:**
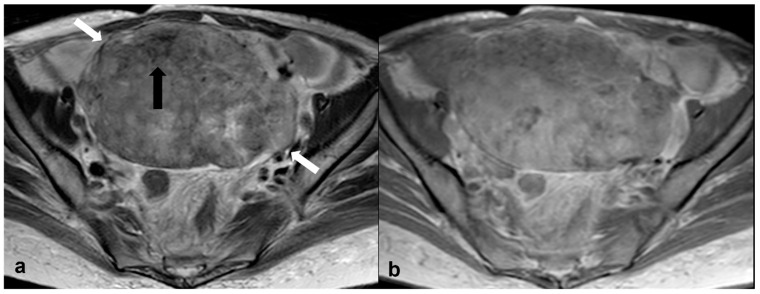
Malignant Brenner tumor. Axial T2 weighted image (**a**) shows a large, predominantly solid mass of moderately high signal intensity (white arrows) with areas of lower T2 signal (black arrow), occupying the pelvis. Axial T1 weighted CE image (**b**) shows mild, heterogeneous enhancement of the mass.

At imaging, their size varies from microscopic to huge; in a series by Moon et al. including eight tumors, the mean size was reported to be 11 cm [75]. In addition, about half of the cases demonstrated extensive amorphous punctuate calcifications [75]. Characteristically, the solid component exhibits markedly low T2 signal intensity on MRI because of dense fibrous stroma [37,75]. Brenner tumors can coexist with mucinous tumors in the same ovary. Findings suggestive of malignancy in a Brenner tumor, include large cystic parts and an inhomogeneous solid component (i.e., with mild enhancement, due to coexistence of fibrous and malignant components [74,75] (Figure 13).

Tip: Predominantly solid ovarian masses with very low (lower than muscle) signal intensity on T2-weighted MR images are indicative of fibroma, Brenner tumor, and, occasionally, fibrothecoma.

#### 2.3.2. Monodermal Teratoma (Struma Ovarii)

Monodermal (specialized) teratomas are rare. They consist mainly of mature cells, most commonly of thyroid origin (struma ovarii) [76]. Struma ovarii tumors may manifest with hyperthyroidism. Malignant transformation, most often to papillary carcinoma, may occur [77].

**Figure 14:**
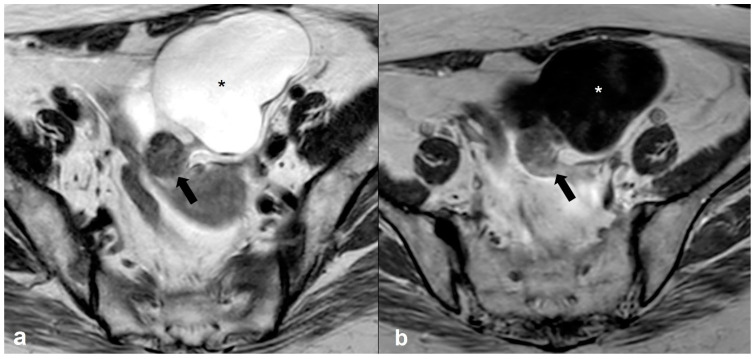
Struma ovarii tumor. Axial T2 weighted image (**a**) shows a predominantly cystic mass (asterisk) with a posteriorly located solid nodule (arrow) in the left ovary showing mild enhancement on the T1 weighted contrast-enhanced image (arrows in (**b**)) and corresponding to ectopic thyroid tissue.

At imaging, they present with a variable mixed solid and cystic component or appear entirely solid (Figure 14). They often coexist with a mature teratoma in which cases the solid component demonstrates avid enhancement. When struma ovarii is suspected, CT may be helpful in narrowing the differential diagnosis, since the solid component is often hyperattenuating on unenhanced images because of the iodine content in the thyroid tissue [78].

#### 2.3.3. Granulosa Cell Tumor

Granulosa cell tumors (GCT) belonging to the sex-cord stromal tumors, are low grade malignant tumors and most are estrogen producing.

Imaging characteristics of GCT vary and may overlap with those of malignant epithelial cell tumors [79]. GCTs are usually present as large (mean size: 10–12 cm), multilocular masses with both solid and cystic components and areas of hemorrhage; they can also be purely solid or cystic. Their most characteristic appearance is that of a solid mass with a spongelike (“Swiss cheese”) appearance; the tumor’s cystic compartments may be hemorrhagic fluid with high T1 signal and fluid-fluid levels [80].

Discriminative features of GCTs from malignant epithelial cell neoplasms include absence of intracystic papillary projections, unilateral location, and confinement to the ovary. Additionally, there is a low incidence of peritoneal disease at diagnosis [79]. Endometrial thickening or endometrioid carcinoma may co-exist due to the estrogenic effect [81].

Tip: Ovarian tumors with estrogen secretion such as, endometrioid carcinoma, granulosa cell tumor, and, occasionally, thecoma or fibrothecoma, can be associated with endometrial hyperplasia or carcinoma.

#### 2.3.4. Lymphoma

Lymphoma of the ovary is usually secondary, occurring as part of systemic disseminated disease; primary lymphoma of the ovary is rare [82].

MRI features of lymphoma of the ovary include the presence of bilateral homogeneous solid masses with mild, homogenous enhancement [83] and rather low T2 signals, due to the presence of myeloperoxidase [84].

**Figure 15:**
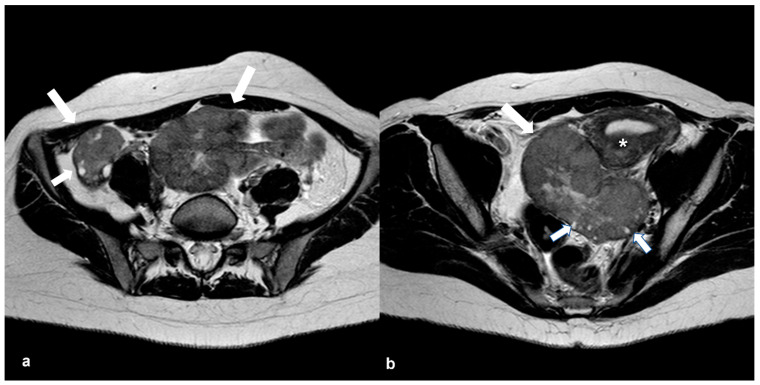
Primary ovarian lymphoma. Axial T2 weighted images (**a**,**b**) show bilateral ovarian enlargement (long arrows) with abnormal stromal signal intensity and peripheral displacement of the ovarian follicles (short arrows). The uterus is displaced to the left side of the pelvis by the enlarged left ovary (asterisk in (**b**)).

Helpful signs for diagnosing ovarian lymphoma are bilaterality, bulky abdominal or pelvic nodal conglomerates, and no ascites. Other characteristic imaging signs include the presence of small peripheral cysts, which correspond to preserved ovarian follicles, and encasement of vessels and bowel by the mass without obstruction [84] (Figure 15).

#### 2.3.5. Metastases

Metastases to the ovary notably occur from tumors of the gastrointestinal tract (colon, appendix, stomach, pancreas), as well as from breast or lung primaries [85].

**Figure 16:**
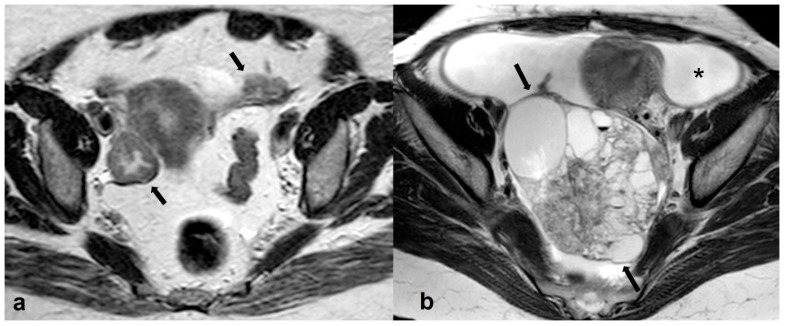
Metastatic ovarian cancer. Axial T2 weighted image (**a**) of a postmenopausal woman with primary gastric cancer. Note the presence of small predominantly solid masses in both ovaries (black arrows). Axial T2 weighted image (**b**) of a premenopausal woman shows a large, mixed cystic, and solid mass in the right adnexa (black arrows) originating from a colon primary. Shown also is a large amount of ascites (asterisk).

They are bilateral in most cases [86]. Imaging manifestations of metastases depend on their site of origin. Predominantly solid metastases usually originate from gastric or breast primaries, while other GI tract metastases (i.e., appendiceal, colorectal, and pancreaticobiliary) often have larger cystic components [87] (Figure 16). At imaging, if bilateral highly vascular ovarian masses measuring less than 10 cm (or for some authors less than 15 cm) are present, metastases should be considered, especially in cases of known history of malignancy or when an extraovarian primary neoplasm with peritoneal carcinomatosis is depicted [88,89]. They are usually associated with heterogeneous hyperintense signal on T2-weighted images because of the variable degree of cystic degeneration; T2-hypointense components may be seen within the metastatic tumor, yet not of lower signal compared to muscle [87].

Radiologists are often challenged to differentiate a primary ovarian mucinous neoplasm from metastasis by an extraovarian mucinous carcinoma, especially when involvement of the ovary is the initial finding, and the primary neoplasm remains unknown. Accurate differential diagnosis is crucial since it highly impacts the right specialty referral and subsequently, treatment.

Imaging signs supporting the diagnosis of mucinous ovarian metastasis over primary borderline or malignant mucinous tumor include (a) size < 10 cm (or for some authors <15 cm), (b) bilateral involvement, and (c) peritoneal dissemination [88,89].

A helpful sign in distinguishing primary borderline and malignant serous tumors from metastases (since they may both present as bilateral tumors with peritoneal spread), is the presence of papillary projections in the former. An imaging feature indicative of ovarian metastases from colorectal tumors is the ‘mille-feuille sign’ which consists of fine, alternating layers of tumor cells and necrosis with a width/length of ≥10/20 mm within the metastatic tumor [90]. Tumor markers may be used as an additional tool by the radiologist to try and reach a diagnosis and help further work-up since high levels of CA-125 are more often observed in primary serous neoplasms, while CEA is often increased in gastrointestinal cancers. It should be noted though that normal assays of tumor markers do not exclude malignancy, because they may not be elevated in small masses or early clinical stages of ovarian cancers.

### 2.4. Adolescence-Germ Cell Tumors

Germ cell tumors represent the second largest category of ovarian neoplasms in the general population; however, they are the most frequently occurring subtype in adolescents and young adults [91]. Histological types include teratoma (mature, immature, and monodermal), dysgerminoma, yolk sac tumor (also known as endodermal sinus tumor), embryonal carcinoma, polyembryoma, and choriocarcinoma. The vast majority of these tumors (95%) are benign; in a number of cases germ cell tumors may be associated with elevated tumor markers including human chorionic gonadotropin (HCG), alfa-fetoprotein (AFP), and the recently introduced embryonic serum microRNAs (MiRNA), which helps in the diagnosis and monitoring of such tumors [92].

#### 2.4.1. Mature Cystic Teratoma (Dermoid)

Mature cystic teratoma is the most common benign ovarian neoplasm in children and adolescents, constituting almost half of all neoplasms in this age population. Bilaterality occurs in 10–25% of cases [93]. Typically, it is asymptomatic; however, complications include torsion (3–16%), rupture (1–4%) and less likely infection (1%), and malignant transformation (1–2%) [94].

Imaging characteristics of a mature teratoma include presence of a cystic, fat-containing mass usually with solid components (i.e., dermoid plug or Rokitansky nodule) [95].

**Figure 17:**
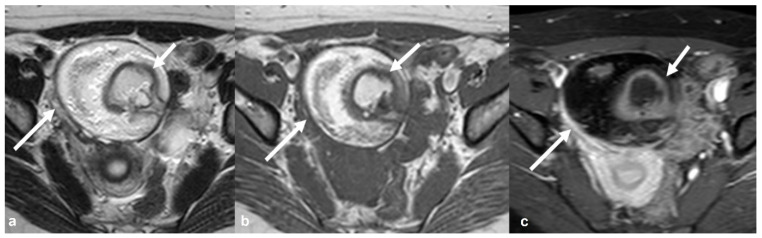
Mature teratoma. Axial T2 weighted image (**a**) shows a large, high signal intensity mass (long white arrow) in the right side of the pelvis. The mass demonstrates high signal intensity on corresponding axial T1 weighted image (**b**) and significant signal drop on T1 weighted FS CE image (**c**), typical of fatty content (long white arrow). Shown also is the typical fat-containing intra-tumoral Rokitansky nodule (short arrow in (**a**) and (**b**)) with peripheral contrast enhancement (short arrow in (**c**)).

At MRI, detection of fatty elements (i.e., high T1 and T2 signal and suppression of signal on fat-suppressed images) is virtually pathognomonic for teratoma. T1-weighted images with saturation of fat is the key sequence for discriminating between fat and hemorrhage (which remains T1 hyperintense). The fat-containing mass shows chemical shift artifact in a significant number of cases (62–87%) [96]. In 25–33% of cases, fat may not be detected within the mass and mature cystic teratomas may, thus, resemble cystic epithelial tumors. In such cases, the wall of the cyst should be carefully inspected for the identification of small T1 and T2 hyperintense foci. Loss of signal intensity on opposed-phase MRI, may assist the diagnosis of intralesional fat [96] (Figure 17). Moreover, DWI may be useful since almost all mature cystic teratomas, even the lipid poor, are known to have a keratinoid component which shows restricted diffusion [97].

Other imaging characteristics of mature teratoma depend on its histologic composition and the presence of sebum, hair, teeth/calcification (in 31% of cases, presenting with low signal intensity at both T1 and T2 weighted images), bone, or cartilage [96].

The solid component of a benign dermoid cyst may show a TIC type 1, 2, or even 3 on DCE which is related to the specific content of the solid tissue of the lesion, without necessarily indicating malignancy (e.g., thyroidal tissue) [98]. Typically, a Rokitansky nodule shows peripheral enhancement, and the lesion is classified as O-RADS MRI 2; however, the presence of large enhancing components within the dermoid, with irregular margins particularly when there is invasion of the cystic wall may indicate malignancy and the lesion is then classified as O-RADS 4 [17]. Most malignant transformations of teratomas (>80%) are of squamous cell histology (SCCs) arising from the ectoderm; less frequently, malignant transformation to carcinoid tumors or adenocarcinomas may occur [99]. Apart from arising in a cystic teratoma, ovarian SCC has been associated with prolonged exposure to various carcinogens and high-risk human papillomavirus (HPV) infection [100].

#### 2.4.2. Immature Teratoma

Immature teratoma is the second most common malignant germ cell tumor in children and adolescents, accounting for 10–20% of all ovarian malignancies in girls younger than 20 years [28]. It is often associated with elevated serum AFP levels (33–65%) [93].

**Figure 18:**
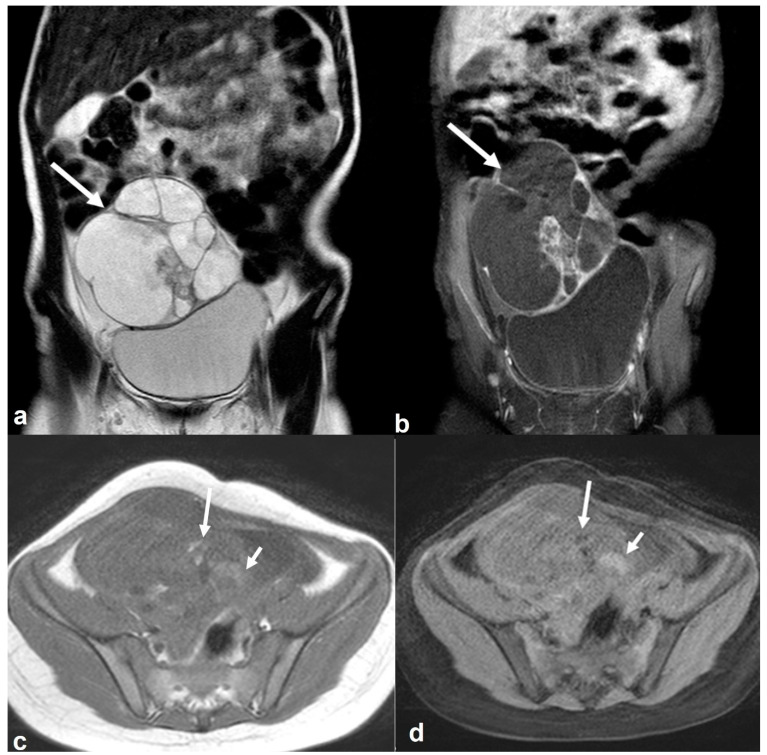
Immature teratoma in a 7-year-old girl. Coronal T2 weighted image (**a**) shows a large cystic right-sided pelvic mass (white arrow) with internal septations and smaller solid components which are enhanced on the T1 weighted FS CE image ((**b**), white arrow). Axial T1 weighted image (**c**) shows tiny foci of high signal intensity (long arrow) with signal loss on the T1 weighted FS image ((**d**), long arrow). Note also a few hemorrhagic foci with high T1 signal intensity on both T1 weighted images (short arrows in (**c**,**d**)).

At MRI, in contrast to benign mature teratomas, it tends to be unilateral and larger, more heterogeneous, with more solid elements which enhance, only small, scattered foci of fat and cystic components with usually simple fluid content. In addition, the calcification pattern seems to differ, since calcifications in immature teratoma are irregular and multiple while in mature tumors they are coarsened or toothlike [96,101] (Figure 18).

Tip: Calcifications may be seen in mature or immature teratoma, Brenner tumor, and less commonly, fibrothecoma of the ovary.

Tip: The presence of fat in an ovarian lesion is virtually pathognomonic of a teratoma. Mature cystic teratomas have an increased number of cystic elements and coarse calcifications, and the solid component presents as a Rokitansky nodule. Immature teratomas contain larger amounts of solid tissue, small foci of fat, and scattered calcifications. Although histological diagnosis cannot be reached with imaging, the suspicion of an immature teratoma can certainly be raised.

#### 2.4.3. Dysgerminoma

Dysgerminoma is the most common malignant germ cell tumor in children and adolescents. Predisposing factors include gonadal dysgenesis, abnormal gonads (gonadoblastoma), and chromosomal syndromes (e.g., Turner syndrome) [102]. Tumor markers, such as LDH and ALP or, rarely β-hCG, are elevated.

Unlike immature teratoma, dysgerminoma may involve both ovaries in a small number of cases (10–15%) and may occasionally spread to the retroperitoneal lymph nodes [102].

**Figure 19:**
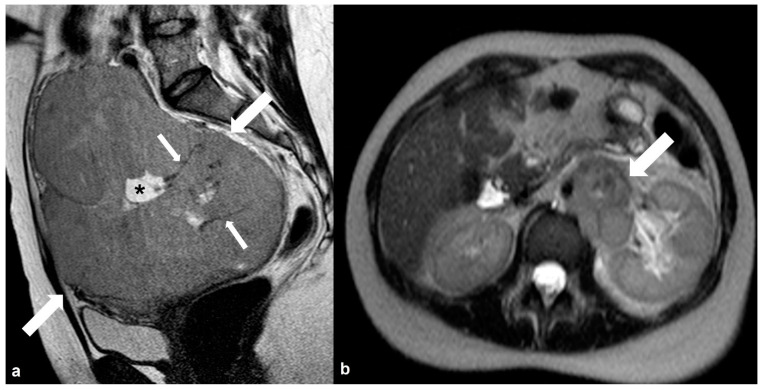
Dygerminoma. Axial T2 weighted image (**a**) shows a predominantly solid ovarian mass (large arrows) with thin low signal intensity internal septa (thin arrows) and cystic/necrotic area (asterisk). T2 weighted image (**b**) of the same patient at the level of the renal hilum shows multiple enlarged left paraaortic lymph nodes (arrow).

Dysgerminoma is typically seen as a large, lobulated predominantly solid mass with internal fibrovascular septa. Because of their fibrous nature, the septa demonstrate low T2 signal intensity and intense, homogenous enhancement [103]. Necrosis, hemorrhage, or speckled calcifications are less frequently seen within the tumor (Figure 19).

Note that, in contrast to other fibrous ovarian tumors (i.e., Brenner tumor and fibroma/fibrothecoma), the T2 signal intensity of fibrous tissue in dysgerminomas is not lower but slightly hyperintense to muscle [104].

### 2.5. Pregnancy

#### 2.5.1. Corpus Luteum Cysts/Theca Lutein Cysts

Corpus luteum cysts, accounting for 13–17% of cystic, pregnancy-related adnexal masses, result from failure of involution of the corpus luteum. The corpus luteum normally forms after ovulation and produces progesterone during the first 8–9 weeks until the placenta takes over [105]. At MRI, corpus luteum cysts appear with variable signal intensity, ragged internal walls, and avid peripheral enhancement [33].

**Figure 20:**
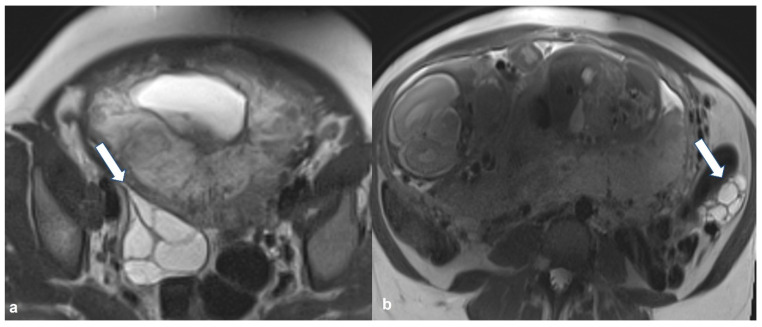
Hyperreactio luteinalis (theca lutein cysts) in third-trimester pregnancy. Axial T2 weighted images shows bilateral, multicystic ovarian masses with a spoke-wheel appearance (arrows in (**a**,**b**)). No solid tissue is present.

Hyperreactio luteinalis (theca lutein cysts) is a rare condition caused by increased levels of β-hCG and manifests as bilateral, multicystic ovarian masses (spoke-wheel appearance), which can mimic ovarian hyperstimulation syndrome or mucinous borderline tumors (Figure 20). It is highly associated with gestational trophoblastic disease and only rarely seen in normal uncomplicated pregnancies [106].

Luteoma of pregnancy is a rare, non-neoplastic ovarian lesion consisting of proliferating luteinized stromal cells, which under the influence of β-hCG, replace normal ovarian parenchyma. As it is a purely solid mass, its discrimination from solid ovarian neoplasms of stromal origin based on imaging is virtually impossible; however, these tumors are usually associated with androgen secretion which may induce maternal and female fetus virilization. If there is suspicion of ovarian luteoma, intervention is not recommended since these lesions spontaneously regress during the early postpartum period [107].

#### 2.5.2. Decidualized Endometrioma

During pregnancy, the ectopic endometrium is characterized by increasing glandular epithelial secretion, stromal vascularity, and oedema due to increased progesterone levels, a change defined as decidualization [108]. At imaging, decidualized endometrioma often appears as a cystic mass with hemorrhagic fluid and a variable amount of enhancing solid component, which can mimic mucinous borderline neoplasms [35,109]. However, decidualized nodules are usually smaller, with signal intensity similar to that of normal placenta, demonstrating higher signal intensity on T2-weighted images and no restricted diffusion on DWI compared to ovarian cancers [110].

#### 2.5.3. Epithelial Ovarian Cancer

**Figure 21:**
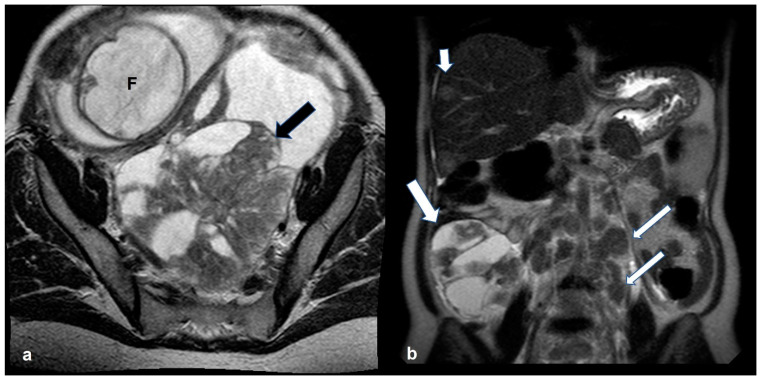
Bilateral high grade epithelial ovarian cancer in a 26 gestational week pregnant woman with twins. Axial T2 weighted image (**a**) shows a mixed cystic-solid left ovarian mass (black arrow). Coronal T2 weighted image (**b**) of the same patient shows a mixed cystic-solid mass in the contralateral ovary (thick white arrow). Shown also are enlarged left paraaortic lymph nodes (thin arrows) and hepatic metastases (short arrows). F: fetus.

Imaging appearances of ovarian cancer in pregnant women do not differ from those in the general population. Evaluation of size and morphologic features can help in distinguishing benign from malignant lesions and the established US and MRI scoring systems can be applied. However, an important limitation of the O-RADS MRI classification system in the gravid population is that gadolinium administration is strongly discouraged for the characterization of a lesion’s solid component since there are still safety issues regarding the effect of paramagnetic contrast media on the fetus [111,112] (Figure 21).

### 2.6. Non-Ovarian Masses

#### 2.6.1. Mucinous Rectosigmoid Cancer

**Figure 22:**
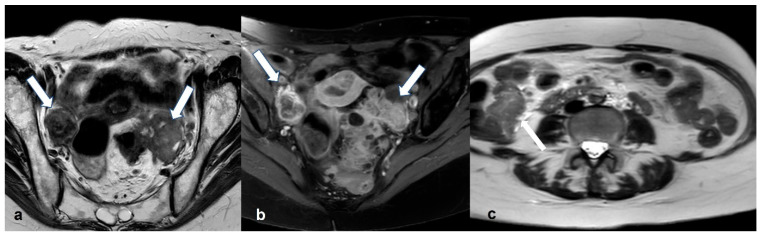
Bilateral ovarian metastases (Krukenberg tumors) from colon cancer. Τ2 weighted axial image (**a**) shows bilateral ovarian soft-tissue masses ((**a**),arrows) exhibiting inhomogeneous enhancement on corresponding T1 weighted FS CE image ((**b**), arrows). Axial T2 weighted image (**c**) of the upper abdomen of the same patient shows extensive circumferential wall thickening of the ascending colon (arrows).

Sigmoid colon adenocarcinoma typically invades the bowel circumferentially narrowing its lumen. At MRI, tumor enhancement and restricted diffusion are common findings. Occasionally, a primary colonic cancer can directly invade or metastasize to the ovaries, making it challenging to accurately identify its origin (Figure 22). Another ambitious task is differentiating between the mucinous subtype of rectal cancer and primary mucinous ovarian neoplasms, since in the case of a large mucin-containing mass, it can be difficult to establish the primary site [113].

#### 2.6.2. Appendiceal Mucocele—PMP

Mucocele of the appendix presents as a distended appendix with mucinous content. It is more commonly seen in middle-aged women and in most cases, it is incidentally found [114]. Underlying histology of an appendiceal mucocele may be that of a simple retention cyst, mucosal hyperplasia, cystadenoma, and cystadenocarcinoma. On MRI, a tubular structure communicating with the base of the caecum with high T2 signal fluid content will be seen [115]. If there is irregular thickening of the wall of the dilated appendix or enhancing nodules cystadenocarcinoma should be suspected.

Accurate preoperative diagnosis and differentiation from mucinous ovarian neoplasms is important in order to avoid rupture during surgery and subsequent pseudomyxoma peritonei formation (i.e., mucinous implants throughout the peritoneal cavity) [116]. On MRI, pseudomyxoma peritonei deposits manifest as low T1 and high T2 foci displacing bowel loops centrally and encasing the bowel lumen, causing bowel obstruction. Frequently, large, complex cystic metastases can also be identified in the ovaries. Characteristically, PMP results in scalloped appearance of the liver and spleen, a useful sign to distinguish mucinous implants from loculated ascites [117].

Tip: PMP is more often the result of a ruptured mucinous adenocarcinoma of the appendix—rupture of mucinous ovarian neoplasms rarely occurs.

#### 2.6.3. Liposarcoma/Schwannoma

Sometimes, distinguishing a large ovarian mass that extends in the upper abdominal spaces from a primary retroperitoneal tumor can be challenging. These tumors are usually of mesenchymal (leiomyoma, sarcoma, solitary fibrous tumor) or neurogenic origin (schwannomas, neurofibromas).

To narrow the differential diagnosis, the first step is to assess the presence of intralesional macroscopic fat. Increased signal intensity on T1-weighted images and signal loss after fat saturation is indicative of a fat-containing lesion. If a fatty mass presents with irregular and ill-defined borders, then the diagnosis of liposarcoma should be considered. Liposarcomas are the most common type of retroperitoneal sarcomas [118]. They typically occur in the 5th and 6th decades of life, mostly in females. At imaging, three distinctive patterns are recognized: mixed, solid, and pseudocystic. The most frequent is the mixed pattern, consisting of a fatty mass with soft-tissue component(s) that usually displaces adjacent organs. Well-differentiated liposarcomas contain an increased amount of fat, whereas high-grade liposarcomas are associated with large soft-tissue components, with appearances similar to those of other sarcomas [119].

If the mass appears predominantly solid or has a myxoid component, one should consider a tumor of neurogenic origin. Schwannoma is a benign, encapsulated tumor of neurogenic origin, commonly seen in young to middle-aged women [120]. On MRI, a low T1 and high T2 signal lesion is seen eccentrically located to a nerve. Areas of cystic degeneration, a pseudocapsule, calcifications, or hemorrhagic foci may also be seen.

Several imaging signs have been described and are associated with benign neurogenic tumors [120]. These include: (a) the fat split sign (a thin rim of high T1 signal corresponding to fat around the lesion), (b) the target sign (peripheral high T2 signal myxoid material and central low T2 signal fibrous component), and (c) the fascicular sign (multiple T2 hypointense ring-like structures surrounded by high T2 signal, representing the fascicular bundles within the nerves).

Ancient schwannoma is a rare benign variant characterized by degenerative changes. On imaging, these tumors appear more heterogeneous compared to a typical schwannoma and due to their cystic component, are often misdiagnosed as malignant tumors. Helpful imaging signs in the differential diagnosis include a smooth enhancing fibrous capsule and degenerative areas with enhancing circumference [121].

**Figure 23:**
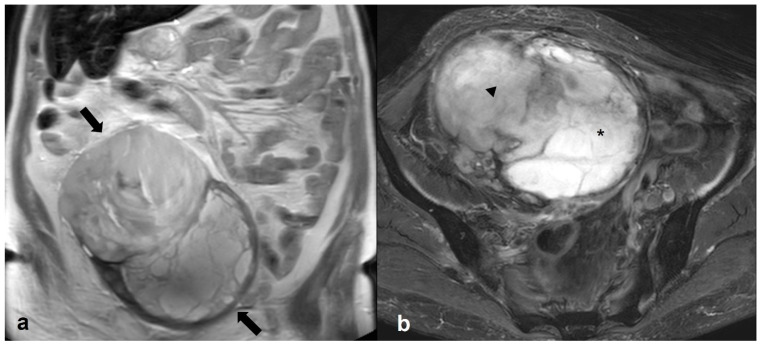
Malignant peripheral nerve sheath tumor (MPNST). Coronal T2 weighted image (**a**) shows a large, encapsulated pelvic mass extending in the upper abdomen (arrows). On the axial T2 weighted FS image (**b**), the mass consists of a high signal intensity, lobulated component on the left (asterisk) and a lower signal intensity component on the right (arrowhead).

Their malignant counterpart, MPNST (Malignant Peripheral Nerve Sheath Tumor), is extremely uncommon and rarely occurs in the retroperitoneum. Peripheral enhancement with non-cystic appearance and heterogeneous enhancement may suggest the diagnosis. Other helpful imaging features to differentiate MPNST from BPNST include: (1) size > 5 cm with ill-defined margins, (2) peritumoral edema, (3) intra-tumoral lobulation, (4) absence of target sign, and (5) bone destruction [122] (Figure 23).

## 3. Conclusions

Accurate differential diagnosis of suspicious pelvic masses based on imaging is crucial since it largely affects clinical management. MRI, with its superior contrast resolution and tissue characterization, can be a useful tool for radiologists. Introduction of 3T magnets in daily clinical routine and application of the O-RADS diagnostic criteria may further familiarize radiologists with the appearances of ovarian cancer and other pelvic diseases that may act as mimickers, increasing their confidence in establishing the site of origin and providing the most likely diagnosis.

## Data Availability

All imaging data were provided by the Department of Radiology, School of Medicine, National and Kapodistrian University of Athens, Aretaieion Hospital, Athens, Greece. These data can be available upon request, while they are not fully uploaded to publicly accessible links due to the General Data Protection Regulation (GDPR) policy of the Hospital.

## References

[1] Kurman R., Carcanjiu M., Herrington S., Young R. (2014). Tumours of the Ovary. World Health Organization Classification of Tumours of the Female Reproductive Organs.

[2] Huang J., Chan W.C., Ngai C.H., Lok V., Zhang L., Lucero-Prisno D.E., Xu W., Zheng Z.-J., Elcarte E., Withers M. (2022). Worldwide Burden, Risk Factors, and Temporal Trends of Ovarian Cancer: A Global Study. Cancers.

[3] Timmerman D., Van Calster B., Testa A.C., Guerriero S., Fischerova D., Lissoni A.A., Van Holsbeke C., Fruscio R., Czekierdowski A., Jurkovic D. (2010). Ovarian Cancer Prediction in Adnexal Masses Using Ultrasound-Based Logistic Regression Models: A Temporal and External Validation Study by the IOTA Group. Ultrasound Obstet. Gynecol..

[4] Van Holsbeke C., Daemen A., Yazbek J., Holland T.K., Bourne T., Mesens T., Lannoo L., Boes A.-S., Joos A., Van De Vijver A. (2010). Ultrasound Experience Substantially Impacts on Diagnostic Performance and Confidence When Adnexal Masses Are Classified Using Pattern Recognition. Gynecol. Obstet. Investig..

[5] Adusumilli S., Hussain H.K., Caoili E.M., Weadock W.J., Murray J.P., Johnson T.D., Chen Q., Desjardins B. (2006). MRI of Sonographically Indeterminate Adnexal Masses. Am. J. Roentgenol..

[6] Sadowski E.A., Paroder V., Patel-Lippmann K., Robbins J.B., Barroilhet L., Maddox E., McMahon T., Sampene E., Wasnik A.P., Blaty A.D. (2018). Indeterminate Adnexal Cysts at US: Prevalence and Characteristics of Ovarian Cancer. Radiology.

[7] Zhang X., Mao Y., Zheng R., Zheng Z., Huang Z., Huang D., Zhang J., Dai Q., Zhou X., Wen Y. (2014). The Contribution of Qualitative CEUS to the Determination of Malignancy in Adnexal Masses, Indeterminate on Conventional US—A Multicenter Study. PLoS ONE.

[8] Karaosmanoglu D., Karcaaltincaba M., Karcaaltincaba D., Akata D., Ozmen M. (2009). MDCT of the Ovarian Vein: Normal Anatomy and Pathology. Am. J. Roentgenol..

[9] Lee J.H., Jeong Y.K., Park J.K., Hwang J.C. (2003). “Ovarian Vascular Pedicle” Sign Revealing Organ of Origin of a Pelvic Mass Lesion on Helical CT. Am. J. Roentgenol..

[10] Arikawa S., Uchida M., Shinagawa M., Tohnan T., Hayabuchi N. (2006). Significance of the “Beak Sign” in the Differential Diagnosis of Uterine Lipoleiomyoma from Ovarian Dermoid Cyst. Kurume Med. J..

[11] Kim J.C., Kim S.S., Park J.Y. (2000). “Bridging Vascular Sign” in the MR Diagnosis of Exophytic Uterine Leiomyoma. J. Comput. Assist. Tomogr..

[12] Sahin H., Panico C., Ursprung S., Simeon V., Chiodini P., Frary A., Carmo B., Smith J., Freeman S., Jimenez-Linan M. (2021). Non-contrast MRI can accurately characterize adnexal masses: A retrospective study. Eur. Radiol..

[13] Lee S.I., Kang S.K. (2023). MRI Improves the Characterization of Incidental Adnexal Masses Detected at Sonography. Radiology.

[14] Liu B., Liao J., Gu W., Wang J., Li G., Wang L. (2021). ADNEX Model-Based Diagnosis of Ovarian Cancer Using MRI Images. Contrast Media Mol. Imaging.

[15] Forstner R., Thomassin-Naggara I., Cunha T.M., Kinkel K., Masselli G., Kubik-Huch R., Spencer J.A., Rockall A. (2017). ESUR Recommendations for MR Imaging of the Sonographically Indeterminate Adnexal Mass: An Update. Eur. Radiol..

[16] Jeong Y.-Y., Outwater E.K., Kang H.K. (2000). Imaging Evaluation of Ovarian Masses. RadioGraphics.

[17] Thomassin-Naggara I., Poncelet E., Jalaguier-Coudray A., Guerra A., Fournier L.S., Stojanovic S., Millet I., Bharwani N., Juhan V., Cunha T.M. (2020). Ovarian-Adnexal Reporting Data System Magnetic Resonance Imaging (O-RADS MRI) Score for Risk Stratification of Sonographically Indeterminate Adnexal Masses. JAMA Netw. Open.

[18] Kang S.K., Reinhold C., Atri M., Benson C.B., Bhosale P.R., Jhingran A., Lakhman Y., Maturen K.E., Nicola R., Pandharipande P.V. (2018). ACR Appropriateness Criteria for Staging And Follow-Up of Ovarian Cancer. J. Am. Coll. Radiol..

[19] Kobal B., Noventa M., Cvjeticanin B., Barbic M., Meglic L., Herzog M., Bordi G., Vitagliano A., Saccardi C., Skof E. (2018). Primary Debulking Surgery versus Primary Neoadjuvant Chemotherapy for High Grade Advanced Stage Ovarian Cancer: Comparison of Survivals. Radiol. Oncol..

[20] McEvoy S.H., Nougaret S., Abu-Rustum N.R., Vargas H.A., Sadowski E.A., Menias C.O., Shitano F., Fujii S., Sosa R.E., Escalon J.G. (2017). Fertility-Sparing for Young Patients with Gynecologic Cancer: How MRI Can Guide Patient Selection Prior to Conservative Management. Abdominal Radiology.

[21] Colombo N., Ledermann J.A. (2021). Updated Treatment Recommendations for Newly Diagnosed Epithelial Ovarian Carcinoma from the ESMO Clinical Practice Guidelines. Ann. Oncol..

[22] Mutch D.G., Prat J. (2014). 2014 FIGO Staging for Ovarian, Fallopian Tube and Peritoneal Cancer. Gynecol. Oncol..

[23] Timmerman D., Testa A.C., Bourne T., Ameye L., Jurkovic D., Van Holsbeke C., Paladini D., Van Calster B., Vergote I., Van Huffel S. (2008). Simple Ultrasound-Based Rules for the Diagnosis of Ovarian Cancer. Ultrasound Obstet. Gynecol..

[24] Viora E., Piovano E., Baima Poma C., Cotrino I., Castiglione A., Cavallero C., Sciarrone A., Bastonero S., Iskra L., Zola P. (2020). The ADNEX Model to Triage Adnexal Masses: An External Validation Study and Comparison with the IOTA Two-Step Strategy and Subjective Assessment by an Experienced Ultrasound Operator. Eur. J. Obstet. Gynecol. Reprod. Biol..

[25] Amor F., Vaccaro H., Alcázar J.L., León M., Craig J.M., Martinez J. (2009). Gynecologic Imaging Reporting and Data System. J. Ultrasound Med..

[26] Andreotti R.F., Timmerman D., Strachowski L.M., Froyman W., Benacerraf B.R., Bennett G.L., Bourne T., Brown D.L., Coleman B.G., Frates M.C. (2020). O-RADS US Risk Stratification and Management System: A Consensus Guideline from the ACR Ovarian-Adnexal Reporting and Data System Committee. Radiology.

[27] Low R.N., Barone R.M., Lucero J. (2015). Comparison of MRI and CT for Predicting the Peritoneal Cancer Index (PCI) Preoperatively in Patients Being Considered for Cytoreductive Surgical Procedures. Ann. Surg. Oncol..

[28] Northridge J.L. (2020). Adnexal Masses in Adolescents. Pediatr. Ann..

[29] Janssen C.L., Littooij A.S., Fiocco M., Huige J.C.B., de Krijger R.R., Hulsker C.C.C., Goverde A.J., Zsiros J., Mavinkurve-Groothuis A.M.C. (2021). The Diagnostic Value of Magnetic Resonance Imaging in Differentiating Benign and Malignant Pediatric Ovarian Tumors. Pediatr. Radiol..

[30] Webb K.E., Sakhel K., Chauhan S.P., Abuhamad A.Z. (2015). Adnexal Mass during Pregnancy: A Review. Am. J. Perinatol..

[31] Telischak N.A., Yeh B.M., Joe B.N., Westphalen A.C., Poder L., Coakley F.V. (2008). MRI of Adnexal Masses in Pregnancy. Am. J. Roentgenol..

[32] Botha M.H., Rajaram S., Karunaratne K. (2018). Cancer in Pregnancy. Int. J. Gynecol. Obstet..

[33] Yacobozzi M., Nguyen D., Rakita D. (2012). Adnexal Masses in Pregnancy. Semin. Ultrasound CT MRI.

[34] Bourgioti C., Konidari M., Gourtsoyianni S., Moulopoulos L.A. (2021). Imaging during Pregnancy: What the Radiologist Needs to Know. Diagn. Interv. Imaging.

[35] Yin M., Wang T., Li S., Zhang X., Yang J. (2022). Decidualized Ovarian Endometrioma Mimicking Malignancy in Pregnancy: A Case Report and Literature Review. J. Ovarian Res..

[36] Murase E., Siegelman E.S., Outwater E.K., Perez-Jaffe L.A., Tureck R.W. (1999). Uterine Leiomyomas: Histopathologic Features, MR Imaging Findings, Differential Diagnosis, and Treatment. RadioGraphics.

[37] Jung S.E., Lee J.M., Rha S.E., Byun J.Y., Jung J.I., Hahn S.T. (2002). CT and MR Imaging of Ovarian Tumors with Emphasis on Differential Diagnosis. RadioGraphics.

[38] Seidman J., Russell P., Kurman R. (2002). Surface Epithelial Tumors of the Ovary. Blaustein’s Pathology of the Female Genital Tract.

[39] Thomassin-Naggara I., Aubert E., Rockall A., Jalaguier-Coudray A., Rouzier R., Daraï E., Bazot M. (2013). Adnexal Masses: Development and Preliminary Validation of an MR Imaging Scoring System. Radiology.

[40] Boger-Megiddo I., Weiss N.S. (2005). Histologic Subtypes and Laterality of Primary Epithelial Ovarian Tumors. Gynecol. Oncol..

[41] Hart W.R. (2005). Borderline Epithelial Tumors of the Ovary. Mod. Pathol..

[42] Folkins A.K., Longacre T.A. (2019). Low-Grade Serous Neoplasia of the Female Genital Tract. Surg. Pathol. Clin..

[43] Bazot M., Haouy D., Daraï E., Cortez A., Dechoux-Vodovar S., Thomassin-Naggara I. (2013). Is MRI a Useful Tool to Distinguish between Serous and Mucinous Borderline Ovarian Tumours?. Clin. Radiol..

[44] Outwater E.K., Huang A.B., Dunton C.J., Talerman A., Capuzzi D.M. (1997). Papillary Projections in Ovarian Neoplasms: Appearance on MRI. J. Magn. Reson. Imaging.

[45] Marko J., Marko K.I., Pachigolla S.L., Crothers B.A., Mattu R., Wolfman D.J. (2019). Mucinous Neoplasms of the Ovary: Radiologic-Pathologic Correlation. RadioGraphics.

[46] Peres L.C., Cushing-Haugen K.L., Köbel M., Harris H.R., Berchuck A., Rossing M.A., Schildkraut J.M., Doherty J.A. (2019). Invasive Epithelial Ovarian Cancer Survival by Histotype and Disease Stage. JNCI J. Natl. Cancer Inst..

[47] Okada S., Ohaki Y., Inoue K., Kawamura T., Hayashi T., Kato T., Kumazaki T. (2005). Calcifications in Mucinous and Serous Cystic Ovarian Tumors. J. Nippon. Med. Sch..

[48] Zhao S.H., Qiang J.W., Zhang G.F., Wang S.J., Qiu H.Y., Wang L. (2014). MRI in Differentiating Ovarian Borderline from Benign Mucinous Cystadenoma: Pathological Correlation. J. Magn. Reson. Imaging.

[49] Okamoto Y., Tanaka Y.O., Tsunoda H., Yoshikawa H., Minami M. (2007). Malignant or Borderline Mucinous Cystic Neoplasms Have a Larger Number of Loculi than Mucinous Cystadenoma: A Retrospective Study with MR. J. Magn. Reson. Imaging.

[50] Ferreira C.R., Carvalho J.P., Soares F.A., Siqueira S.A.C., Carvalho F.M. (2008). Mucinous Ovarian Tumors Associated with Pseudomyxoma Peritonei of Adenomucinosis Type: Immunohistochemical Evidence That They Are Secondary Tumors. Int. J. Gynecol. Cancer.

[51] Fadare O., Parkash V. (2019). Pathology of Endometrioid and Clear Cell Carcinoma of the Ovary. Surg. Pathol. Clin..

[52] Watson P., Bützow R., Lynch H.T., Mecklin J.-P., Järvinen H.J., Vasen H.F.A., Madlensky L., Fidalgo P., Bernstein I. (2001). The Clinical Features of Ovarian Cancer in Hereditary Nonpolyposis Colorectal Cancer. Gynecol. Oncol..

[53] Matias-Guiu X., Stewart C.J.R. (2018). Endometriosis-Associated Ovarian Neoplasia. Pathology.

[54] Tanaka Y.O., Yoshizako T., Nishida M., Yamaguchi M., Sugimura K., Itai Y. (2000). Ovarian Carcinoma in Patients with Endometriosis. Am. J. Roentgenol..

[55] Tanaka Y.O., Okada S., Yagi T., Satoh T., Oki A., Tsunoda H., Yoshikawa H. (2010). MRI of Endometriotic Cysts in Association With Ovarian Carcinoma. Am. J. Roentgenol..

[56] van Niekerk C.C., Bulten J., Vooijs G.P., Verbeek A.L.M. (2010). The Association between Primary Endometrioid Carcinoma of the Ovary and Synchronous Malignancy of the Endometrium. Obstet. Gynecol. Int..

[57] Matsuura Y., Robertson G., Marsden D.E., Kim S.-N., Gebski V., Hacker N.F. (2007). Thromboembolic Complications in Patients with Clear Cell Carcinoma of the Ovary. Gynecol. Oncol..

[58] Savvari P., Peitsidis P., Alevizaki M., Dimopoulos M.-A., Antsaklis A., Papadimitriou C.A. (2009). Paraneoplastic Humorally Mediated Hypercalcemia Induced by Parathyroid Hormone-Related Protein in Gynecologic Malignancies: A Systematic Review. Onkologie.

[59] Avesani G., Caliolo G., Gui B., Petta F., Panico C., La Manna V., Moro F., Testa A.C., Scambia G., Manfredi R. (2021). Pearls and Potential Pitfalls for Correct Diagnosis of Ovarian Cystadenofibroma in MRI: A Pictorial Essay. Korean J. Radiol..

[60] Tang Y.Z., Liyanage S., Narayanan P., Sahdev A., Sohaib A., Singh N., Rockall A. (2013). The MRI Features of Histologically Proven Ovarian Cystadenofibromas—An Assessment of the Morphological and Enhancement Patterns. Eur. Radiol..

[61] Jung D.C., Kim S.H., Kim S.H. (2006). MR Imaging Findings of Ovarian Cystadenofibroma and Cystadenocarcinofibroma: Clues for the Differential Diagnosis. Korean J. Radiol..

[62] Chen J., Wang J., Chen X., Wang Y., Wang Z., Li D. (2017). Computed Tomography and Magnetic Resonance Imaging Features of Ovarian Fibrothecoma. Oncol. Lett..

[63] Cho Y.J., Lee H.S., Kim J.M., Joo K.Y., Kim M.-L. (2013). Clinical Characteristics and Surgical Management Options for Ovarian Fibroma/Fibrothecoma: A Study of 97 Cases. Gynecol. Obstet. Invest..

[64] Shinagare A.B., Meylaerts L.J., Laury A.R., Mortele K.J. (2012). MRI Features of Ovarian Fibroma and Fibrothecoma With Histopathologic Correlation. Am. J. Roentgenol..

[65] Zhang H., Zhang G.-F., Wang T.-P., Zhang H. (2013). Value of 3.0 T Diffusion-Weighted Imaging in Discriminating Thecoma and Fibrothecoma from Other Adnexal Solid Masses. J. Ovarian Res..

[66] Chung B.M., bin Park S., Lee J.B., Park H.J., Kim Y.S., Oh Y.J. (2015). Magnetic Resonance Imaging Features of Ovarian Fibroma, Fibrothecoma, and Thecoma. Abdom. Imaging.

[67] Agostinho L., Horta M., Salvador J.C., Cunha T.M. (2019). Benign Ovarian Lesions with Restricted Diffusion. Radiol. Bras..

[68] Tanaka Y.O., Tsunoda H., Kitagawa Y., Ueno T., Yoshikawa H., Saida Y. (2004). Functioning Ovarian Tumors: Direct and Indirect Findings at MR Imaging. RadioGraphics.

[69] Chen H., Liu Y., Shen L., Jiang M., Yang Z., Fang G. (2016). Ovarian Thecoma-Fibroma Groups: Clinical and Sonographic Features with Pathological Comparison. J. Ovarian Res..

[70] Thomassin-Naggara I., Darai E., Bazot M. (2012). Gynecological Pelvic Infection: What Is the Role of Imaging?. Diagn. Interv. Imaging.

[71] Revzin M.V., Mathur M., Dave H.B., Macer M.L., Spektor M. (2016). Pelvic Inflammatory Disease: Multimodality Imaging Approach with Clinical-Pathologic Correlation. RadioGraphics.

[72] Tukeva T.A., Aronen H.J., Karjalainen P.T., Molander P., Paavonen T., Paavonen J. (1999). MR Imaging in Pelvic Inflammatory Disease: Comparison with Laparoscopy and US. Radiology.

[73] Bonde A., Andreazza Dal Lago E., Foster B., Javadi S., Palmquist S., Bhosale P. (2022). Utility of the Diffusion Weighted Sequence in Gynecological Imaging: Review Article. Cancers.

[74] Takeuchi M., Matsuzaki K., Sano N., Furumoto H., Nishitani H. (2008). Malignant Brenner Tumor with Transition from Benign to Malignant Components. J. Comput. Assist. Tomogr..

[75] Moon W.J., Koh B.H., Kim S.K., Kim Y.S., Rhim H.C., Cho O.K., Hahm C.K., Byun J.Y., Cho K.S., Kim S.H. (2000). Brenner Tumor of the Ovary: CT and MR Findings. J. Comput. Assist. Tomogr..

[76] Euscher E.D. (2019). Germ Cell Tumors of the Female Genital Tract. Surg. Pathol. Clin..

[77] Makani S., Kim W., Gaba A.R. (2004). Struma Ovarii with a Focus of Papillary Thyroid Cancer: A Case Report and Review of the Literature. Gynecol. Oncol..

[78] Taylor E.C., Irshaid L., Mathur M. (2020). Multimodality Imaging Approach to Ovarian Neoplasms with Pathologic Correlation. RadioGraphics.

[79] Elsherif S., Bourne M., Soule E., Lall C., Bhosale P. (2020). Multimodality Imaging and Genomics of Granulosa Cell Tumors. Abdom. Radiol..

[80] Zhang H., Zhang H., Gu S., Zhang Y., Liu X., Zhang G. (2018). MR Findings of Primary Ovarian Granulosa Cell Tumor with Focus on the Differentiation with Other Ovarian Sex Cord-Stromal Tumors. J. Ovarian Res..

[81] Fotopoulou C., Savvatis K., Braicu E.-I., Brink-Spalink V., Darb-Esfahani S., Lichtenegger W., Sehouli J. (2010). Adult Granulosa Cell Tumors of the Ovary: Tumor Dissemination Pattern at Primary and Recurrent Situation, Surgical Outcome. Gynecol. Oncol..

[82] Lagoo A.S., Robboy S.J. (2006). Lymphoma of the Female Genital Tract: Current Status. Int. J. Gynecol. Pathol..

[83] Slonimsky E., Korach J., Perri T., Davidson T., Apter S., Inbar Y. (2018). Gynecological Lymphoma. J. Comput. Assist. Tomogr..

[84] Crawshaw J., Sohaib S.A., Wotherspoon A., Shepherd J.H. (2007). Primary Non-Hodgkin’s Lymphoma of the Ovaries: Imaging Findings. Br. J. Radiol..

[85] Agnes A., Biondi A., Ricci R., Gallotta V., D’Ugo D., Persiani R. (2017). Krukenberg Tumors: Seed, Route and Soil. Surg. Oncol..

[86] Lerwill M.F., Young R.H. (2008). Metastatic Tumors of the Ovary. Blaustein’s Pathology of the Female Genital Tract.

[87] Koyama T., Mikami Y., Saga T., Tamai K., Togashi K. (2007). Secondary Ovarian Tumors: Spectrum of CT and MR Features with Pathologic Correlation. Abdom. Imaging.

[88] Khunamornpong S., Suprasert P., Pojchamarnwiputh S., Na Chiangmai W., Settakorn J., Siriaunkgul S. (2006). Primary and Metastatic Mucinous Adenocarcinomas of the Ovary: Evaluation of the Diagnostic Approach Using Tumor Size and Laterality. Gynecol. Oncol..

[89] Xu Y., Yang J., Zhang Z., Zhang G. (2015). MRI for Discriminating Metastatic Ovarian Tumors from Primary Epithelial Ovarian Cancers. J. Ovarian Res..

[90] Kurokawa R., Nakai Y., Gonoi W., Mori H., Tsuruga T., Makise N., Ushiku T., Abe O. (2020). Differentiation between Ovarian Metastasis from Colorectal Carcinoma and Primary Ovarian Carcinoma: Evaluation of Tumour Markers and “Mille-Feuille Sign” on Computed Tomography/Magnetic Resonance Imaging. Eur. J. Radiol..

[91] Talerman A. (2002). Germ Cell Tumors of the Ovary. Blaustein’s Pathology of the Female Genital Tract.

[92] Jezierska M., Gawrychowska A., Stefanowicz J. (2022). Diagnostic, Prognostic and Predictive Markers in Pediatric Germ Cell Tumors—Past, Present and Future. Diagnostics.

[93] Heo S.H., Kim J.W., Shin S.S., Jeong S.I., Lim H.S., Choi Y.D., Lee K.H., Kang W.D., Jeong Y.Y., Kang H.K. (2014). Review of Ovarian Tumors in Children and Adolescents: Radiologic-Pathologic Correlation. RadioGraphics.

[94] bin Park S., Kim J.K., Kim K.-R., Cho K.-S. (2008). Imaging Findings of Complications and Unusual Manifestations of Ovarian Teratomas. RadioGraphics.

[95] Saleh M., Bhosale P., Menias C.O., Ramalingam P., Jensen C., Iyer R., Ganeshan D. (2021). Ovarian Teratomas: Clinical Features, Imaging Findings and Management. Abdom. Radiol..

[96] Outwater E.K., Siegelman E.S., Hunt J.L. (2001). Ovarian Teratomas: Tumor Types and Imaging Characteristics. RadioGraphics.

[97] Nakayama T., Yoshimitsu K., Irie H., Aibe H., Tajima T., Nishie A., Asayama Y., Matake K., Kakihara D., Matsuura S. (2005). Diffusion-Weighted Echo-Planar MR Imaging and ADC Mapping in the Differential Diagnosis of Ovarian Cystic Masses: Usefulness of Detecting Keratinoid Substances in Mature Cystic Teratomas. J. Magn. Reson. Imaging.

[98] Poncelet E., Delpierre C., Kerdraon O., Lucot J.-P., Collinet P., Bazot M. (2013). Value of Dynamic Contrast-Enhanced MRI for Tissue Characterization of Ovarian Teratomas: Correlation with Histopathology. Clin. Radiol..

[99] Kido A., Togashi K., Konishi I., Kataoka M.L., Koyama T., Ueda H., Fujii S., Konishi J. (1999). Dermoid Cysts of the Ovary with Malignant Transformation: MR Appearance. Am. J. Roentgenol..

[100] Verguts J., Amant F., Moerman P., Vergote I. (2007). HPV Induced Ovarian Squamous Cell Carcinoma: Case Report and Review of the Literature. Arch. Gynecol. Obstet..

[101] Yamaoka T., Togashi K., Koyama T., Fujiwara T., Higuchi T., Iwasa Y., Fujii S., Konishi J. (2003). Immature Teratoma of the Ovary: Correlation of MR Imaging and Pathologic Findings. Eur. Radiol..

[102] Laufer M., Goldstein D. (2005). Benign and Malignant Ovarian Masses. Pediatric and Adolescent Gynecology.

[103] Kitajima K., Hayashi M., Kuwata Y., Imanaka K., Sugimura K. (2007). MRI Appearances of Ovarian Dysgerminoma. Eur. J. Radiol. Extra.

[104] Shaaban A.M., Rezvani M., Elsayes K.M., Baskin H., Mourad A., Foster B.R., Jarboe E.A., Menias C.O. (2014). Ovarian Malignant Germ Cell Tumors: Cellular Classification and Clinical and Imaging Features. RadioGraphics.

[105] Schwartz N., Timor-Tritsch I.E., WANG E. (2009). Adnexal Masses in Pregnancy. Clin. Obstet. Gynecol..

[106] van Holsbeke C., Amant F., Veldman J., de Boodt A., Moerman P., Timmerman D. (2009). Hyperreactio Luteinalis in a Spontaneously Conceived Singleton Pregnancy. Ultrasound Obstet. Gynecol..

[107] Cathcart A.M., Nezhat F.R., Emerson J., Pejovic T., Nezhat C.H., Nezhat C.R. (2022). Adnexal Masses during Pregnancy: Diagnosis, Treatment, and Prognosis. Am. J. Obstet. Gynecol..

[108] Barbieri M., Somigliana E., Oneda S., Ossola M.W., Acaia B., Fedele L. (2009). Decidualized Ovarian Endometriosis in Pregnancy: A Challenging Diagnostic Entity. Hum. Reprod..

[109] Takeuchi M., Matsuzaki K., Nishitani H. (2008). Magnetic Resonance Manifestations of Decidualized Endometriomas During Pregnancy. J. Comput. Assist. Tomogr..

[110] Bourgioti C., Preza O., Panourgias E., Chatoupis K., Antoniou A., Nikolaidou M.E., Moulopoulos L.A. (2017). MR Imaging of Endometriosis: Spectrum of Disease. Diagn. Interv. Imaging.

[111] Thomassin-Naggara I., Fedida B., Sadowski E., Chevrier M.-C., Chabbert-Buffet N., Ballester M., Tavolaro S., Darai E. (2017). Complex US Adnexal Masses during Pregnancy: Is Pelvic MR Imaging Accurate for Characterization?. Eur. J. Radiol..

[112] Bourgioti C., Konidari M., Moulopoulos L.A. (2020). Imaging of Gynecologic Malignancy in a Reproductive Age Female. Radiol. Clin. N. Am..

[113] Nougaret S., Nikolovski I., Paroder V., Vargas H.A., Sala E., Carrere S., Tetreau R., Hoeffel C., Forstner R., Lakhman Y. (2019). MRI of Tumors and Tumor Mimics in the Female Pelvis: Anatomic Pelvic Space–Based Approach. RadioGraphics.

[114] Dhage-Ivatury S., Sugarbaker P.H. (2006). Update on the Surgical Approach to Mucocele of the Appendix. J. Am. Coll. Surg..

[115] Van Hooser A., Williams T.R., Myers D.T. (2018). Mucinous Appendiceal Neoplasms: Pathologic Classification, Clinical Implications, Imaging Spectrum and Mimics. Abdom. Radiol..

[116] Carr N.J., Cecil T.D., Mohamed F., Sobin L.H., Sugarbaker P.H., González-Moreno S., Taflampas P., Chapman S., Moran B.J. (2016). A Consensus for Classification and Pathologic Reporting of Pseudomyxoma Peritonei and Associated Appendiceal Neoplasia. Am. J. Surg. Pathol..

[117] Chira R.I., Nistor-Ciurba C.C., Mociran A., Mircea P.A. (2016). Appendicular Mucinous Adenocarcinoma Associated with Pseudomyxoma Peritonei, a Rare and Difficult Imaging Diagnosis. Med. Ultrason..

[118] Nishino M., Hayakawa K., Minami M., Yamamoto A., Ueda H., Takasu K. (2003). Primary Retroperitoneal Neoplasms: CT and MR Imaging Findings with Anatomic and Pathologic Diagnostic Clues. RadioGraphics.

[119] Kransdorf M.J., Bancroft L.W., Peterson J.J., Murphey M.D., Foster W.C., Temple H.T. (2002). Imaging of Fatty Tumors: Distinction of Lipoma and Well-Differentiated Liposarcoma. Radiology.

[120] Hoarau N., Slim K., da Ines D. (2013). CT and MR Imaging of Retroperitoneal Schwannoma. Diagn. Interv. Imaging.

[121] Isobe K., Shimizu T., Akahane T., Kato H. (2004). Imaging of Ancient Schwannoma. Am. J. Roentgenol..

[122] Yu Y., Wu J., Ye J., Chen M. (2016). Radiological Findings of Malignant Peripheral Nerve Sheath Tumor: Reports of Six Cases and Review of Literature. World J. Surg. Oncol..

